# Pediatric Infectious Diseases: Getting Research Evidence into Practice and Generation of New Evidence

**DOI:** 10.3389/fped.2014.00138

**Published:** 2014-12-08

**Authors:** Hans Van Rostenberghe

**Affiliations:** ^1^Department of Paediatrics, Universiti Sains Malaysia, Kubang Kerian, Malaysia

**Keywords:** evidence, infection control, prevention, diagnosis, treatment

If a person who died in the early years of the twentieth century would come back today, he would not believe what he saw, heard, felt, tasted, and smelled. The twentieth century has been a century of so many discoveries and advances, unprecedented in any previous era.

Also, the understanding, the prevention, the diagnosis and treatment of infectious diseases in pediatrics have made major leaps forward in the previous century (1). Eradication of diseases through vaccines has become possible; proper hygienic measures have reduced spread of infections within hospitals tremendously, antibiotics have allowed us to cure diseases that before carried a grave prognosis (1). Evidence based medicine has allowed us to bust some myths of widely practiced treatments and conclusively proven the efficacy of others. Knowledge and technology have grown exponentially and are continuing to do so in the twenty-first century (1).

While the exciting discoveries keep going on, a major challenge ahead of us lies in the effective processing, application, and implementation of all new discoveries of the past and current century. The grand challenge of this new field of Frontiers in Pediatrics will focus on two main areas: getting research evidence in practice and new exciting evidence that could impact clinical practice.

## Getting Research into Practice

There are many areas where creative and innovative approaches are very much needed to give all children in the world a chance to enjoy optimal health and protection from infections, based on evidence created in the last few decades. Below, a few examples of extremely important areas are presented.

### Effective infection control

Very strong evidence exists, since almost 100 years ago, that proper hand hygiene can make a huge difference in the incidence of hospital acquired infections (2, 3). But up to very recently, studies determining the compliance of health care workers to hand hygiene policies showed disturbingly poor levels of compliance: 20, 40, 80%? Articles published in the last year still report 40% pre-intervention and 60% post intervention (4). Why is it not 100%? Simple hand washing or hand rubbing with disinfectant solution are among the most effective ways to control hospital infection, but up to today many places in the world fail to implement this practice effectively (3).

Many approaches have been tried to increase hand hygiene compliance and the success among approaches was variable at the best, with strategies addressing a large number of factors (knowledge, awareness, social, behavior, and more) having the largest effect (5). Very few studies report on prolonged sustainability of interventions. We may need to integrate psychological, behavioral, and management approaches with clinical medicine and education of staff to make this very simple but highly effective method of infection control work for all children in the world. Novel and creative approaches improve practice of infection control within hospitals are definitely among the priority fields of interest for this journal.

Also, outside of the healthcare setting, simple measures to improve the hygiene of children may have a huge impact. Overcrowding has since long been known to be a risk factor for RSV (6). Many of the epidemics of the respiratory viruses are now believed to be due to overcrowding in the houses. In Hong Kong, the higher incidences of RSV were seen in months with hotter weather, when people preferred to stay inside rather than to spend the time outside of the house (7). Contaminated hands are believed to transfer respiratory infections much more effective from one person to another than infected droplets. It is estimated that through proper hand washing each person could reduce his own respiratory infections by more than 60%. RSV transmission is preventable by increasing hand hygiene, by avoiding simple things as passive smoking for the high-risk children (8).

But up to today again, many children are exposed to these simply avoidable risk factors and come down with severe infections that may have been prevented in many ways. Proper health education is often lacking, even in middle and high-income countries. In low-income countries, not only health education is lacking but also the basic facilities to practice proper hygiene may be not available to the majority of families with children. Even though, in our current times, we know about the DNA of human beings and bacteria, we know about protons and bosons and quarks, we have the most advanced technology available, too many children still die in today’s world of infections that were preventable through simple measures.

### Vaccines

Prevention of infection is extremely important and many effective vaccines have been produced since many decades. Major successes have been achieved through immunization. Smallpox is still deemed to be eradicated (9). Major strides have been taken toward a polio free world. Measles has become a rare disease in most middle and high-income countries. Congenital rubella has become a perfectly preventable condition. Tetanus, pertussis diphtheria, and so many other infectious diseases have been controlled in major ways through vaccination (9).

Still there is a large group of people who have become increasingly vocal, spreading untrue health beliefs about the damaging nature of many of the available very effective and highly safe vaccines. Social media have given an extra voice to such groups and if these groups are allowed to spread their influence they may not only jeopardize the health of their own children but also compromise the herd immunity, which is very useful for the small number of children with true contra-indications for vaccination. Clinicians can counteract this (10).

Reports on community intervention programs providing innovative, creative methods of health education on infection prevention are a priority area for this journal.

### Antibiotics

It may be hard today to imagine a world without antibiotics but <100 years ago, no antibiotics were around. Antibiotics have saved numerous lives and they have played perhaps a pivotal role in the success of allopathic medicine. Abuse of antibiotic has led to emerging problems. Of course, some of the side effects can be particularly nasty and lead to death or life long sequelae but a main problem with non-rational use of antibiotics is the emergence of resistant bacterial strains (11). Again evidence is ample but practice lags behind. Up to today, antibiotics are abused in many places. Strict control of antibiotic usage may reduce resistance dramatically (12). Avoidance of strong inducers of resistance such as cephalosporins may have a beneficial effect. Further research is needed. It is hoped that research into rational antibiotic usage and control can be reported in this section of the journal.

### Vector borne diseases and other major infections

Effective vector control is possible for many vector borne diseases. However, diseases as malaria (13) and dengue (14) fever are still endemic in many parts of the world. Again bringing the evidence into practice is lagging behind. HIV and tuberculous, even though to a high-degree preventable, claim still many children’s lives every year worldwide (13).

## New Evidence

While the mammoth task of getting all available evidence into practice for all children in the world is ongoing, of course science does not stop. Plenty of new discoveries are made every year. Exciting new areas of research in pediatric infectious diseases have been explored and many new areas and discoveries will be made over the coming decade.

The editors of this journal will welcome articles reporting contributions to novel ways of preventing, diagnosing, and treating infections. The exponentially evolving fields of genetics, immunology, microbiology, chemistry, and physics combined with extreme advances in technology to the nano level (15) and the huge potential of stem cell therapy (16) seem extremely promising for the medicine of the future as a whole. While it is close to impossible to give a comprehensive overview of all exciting new developments in the field, a few really important ones will be highlighted.

The new vaccines for devastating diseases like dengue fever are certainly among the very relevant developments. Recently, a commercial dengue vaccine was approved by the FDA. It delivers only very partial protection (56% reduction in cases) against dengue fever in the vaccinated children (17). But more important questions remain. Antibodies are well known to play an important role in dengue hemorrhagic shock syndrome (18). Will the acquired partial immunity result in worse rates of the most feared complication of dengue once the children become adults or will it still be protective and beneficial?

Similar questions arise when we talk about RSV (19), even though recent evidence seems to support that active vaccination against RSV may become available in the near future depending on the results of ongoing trials (20). Shall we get better vaccines against tuberculosis any time soon? Is effective safe vaccination against HIV on the horizon (21) and how will it be received among extremist religious groups who imagine they can effectively prevent the disease exclusively through moral teachings and policing?

Another worrying development in the treatment of infectious diseases is the apparent loss of interest from the industry in developing new antibiotics. In the last one decade, there has been an ever decreasing number of new antibiotics coming to the market and being tested (22). This may be related the lucrative and exorbitant profits that can be made through novel cancer treatments and other emerging diseases. Multi-drug-resistant (MDR) organisms (23) are like a sword of Damocles hanging above the future of intensive care if no effective strategies to prevent their development and newer antibiotics become available.

Particularly interesting is that most antibiotics are used for neonates and children off label since economic interest are not in the favor of children (24). Chemoprophylaxis has been studied in a few cases and short beneficial outcomes have been reported by hopeful researchers. But long-term worries about the potential development of MDR organisms remain the main obstacle for implementing these practices (25). Research in these areas will definitely be among the priorities of this journal.

Pediatric infectious diseases will surely benefit in many ways from these rapid advances. These are exciting times. Our bravest imagination may fall short to figure out what the future may bring. It is hoped that this journal will also be a platform where these developments will be published and discussed.

## Conclusion

It is hoped that this journal can contribute in a major way to effective implementation of health interventions that reduce the burden of infection in the community and in the hospital. Exciting new developments in pediatric infectious diseases are anticipated and this will be the place to publish and read it.

## Conflict of Interest Statement

The author declares that the research was conducted in the absence of any commercial or financial relationships that could be construed as a potential conflict of interest. The Field Chief Editor, Professor Antonio Francesco Corno, declares that, despite being affiliated with the same institution as the author, Professor Hans Van Rostenberghe, the review process was handled objectively and no conflict of interest exists.
